# Human expansion precipitates niche expansion for an opportunistic apex predator (*Puma concolor*)

**DOI:** 10.1038/srep39639

**Published:** 2016-12-23

**Authors:** Wynne E. Moss, Mathew W. Alldredge, Kenneth A. Logan, Jonathan N. Pauli

**Affiliations:** 1 Department of Forest & Wildlife Ecology, University of Wisconsin, Madison, Wisconsin 53706, USA; 2 Colorado Parks & Wildlife, Fort Collins, Colorado, 80525, USA

## Abstract

There is growing recognition that developed landscapes are important systems in which to promote ecological complexity and conservation. Yet, little is known about processes regulating these novel ecosystems, or behaviours employed by species adapting to them. We evaluated the isotopic niche of an apex carnivore, the cougar (*Puma concolor*), over broad spatiotemporal scales and in a region characterized by rapid landscape change. We detected a shift in resource use, from near complete specialization on native herbivores in wildlands to greater use of exotic and invasive species by cougars in contemporary urban interfaces. We show that 25 years ago, cougars inhabiting these same urban interfaces possessed diets that were intermediate. Thus, niche expansion followed human expansion over both time and space, indicating that an important top predator is interacting with prey in novel ways. Thus, though human-dominated landscapes can provide sufficient resources for apex carnivores, they do not necessarily preserve their ecological relationships.

The conversion of wildlands to developed habitat is a pervasive threat to native species, and tends to create biotically homogenous communities^1^ differing strongly from their historical norm^2^. Though conservation efforts have traditionally focused on preserving pristine habitat, there is now growing interest in enhancing biodiversity in already transformed ecosystems (e.g. the “New Conservation” movement^3^), including human-dominated landscapes. Indeed, accumulating evidence suggests that urban ecosystems can represent viable habitat for species of conservation importance^4^. Yet, maintaining functional ecological relationships in these novel and transformed systems will be challenging, as they feature community assemblages and interactions that are entirely new and poorly understood^2^.

Large-bodied carnivores have received disproportionate attention for their role as ecosystem regulators, and are often targeted as a means to restore stability to systems altered by human activity^5^. Until recently, it was assumed that only smaller-bodied mesocarnivores could exploit highly developed areas while large carnivores were excluded due to their sensitivity to fragmentation and enhanced conflict with humans^6^. After decades of decline, many large apex carnivores are rebounding in North America and Europe, and they are now increasingly using developed and urban habitats worldwide^7^,^8^,^9^,^10^. To understand the value and function of such ecosystems in global conservation, it is essential to measure how species at the highest trophic levels behave and exploit resources within them.

Though much of our understanding of large carnivore ecology is derived from wildland systems, accumulating evidence suggests that habitat development significantly alters their behaviour and ecology in predictable ways. Due to shifts in prey communities, bottom-up subsidies, and altered risk landscapes in these emerging developed ecosystems, resource use of apex carnivores can differ strongly from historic patterns^11^. Dietary shifts, along with changes in demography^12^ have the potential to alter top-down forcing, with implications for ecosystem stability and resilience^13^. Thus, it has been suggested that apex carnivores in developed ecosystems are returning in name only, possessing a novel ecological niche^14^.

Herein, we provide evidence that an ecologically important and rebounding apex carnivore^15^, the cougar (*Puma concolor*), has recently diversified its resource use and, therefore, is expanding its niche and interacting with novel prey in highly developed ecosystems. Traditionally viewed as wildland specialists reliant on tracts of protected land with high ungulate densities^16^,^17^, cougars are increasingly found utilizing a gradient of human-developed landscapes^18^,^19^,^20^. However, fundamental aspects of their ecology, including survival rates^18^,^21^ and diet^21^,^22^, appear to differ in highly developed landscapes. To understand how rapid and extensive this dietary shift is, and how cougar-prey interactions may change within these novel ecosystems, we analysed the isotopic signatures of three cougar populations in Colorado, USA: a contemporary wildland population, a contemporary population in an urban interface, and a population in that same urban interface 25 years prior. By modelling isotopic niche over broad spatiotemporal scales, we detected changes in resource use over space and time, including higher use of exotic and synanthropic prey in today’s urban interface. Over the past 25 years, cougar populations near human development expanded their diet, from near specialization on native herbivore prey to a more generalist diet. Thus, the interactions between cougars and their prey appear to shift in human-dominated landscapes, with implications for ecosystem functioning.

## Results

**Figure 1:**
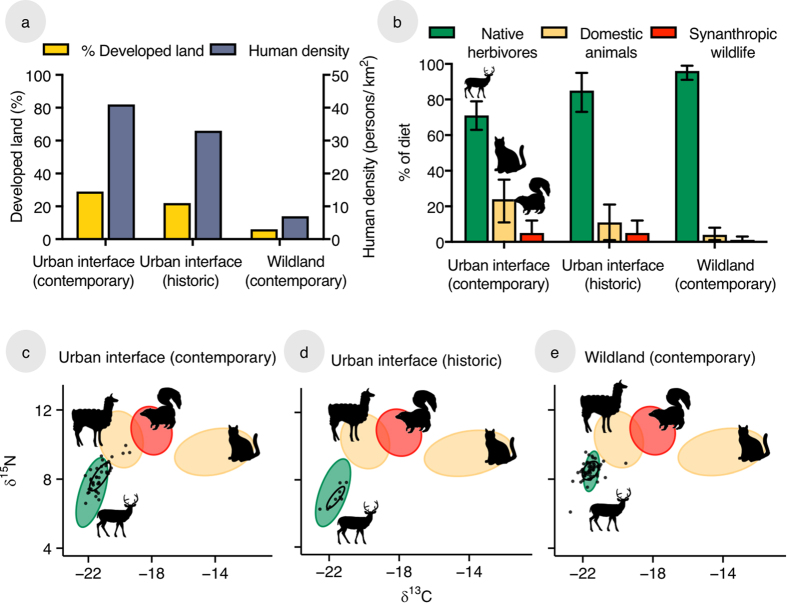
Cougar niche varies with anthropogenic change. (**a**) Sites differed in land use (% of study area classified as developed; primary axis) and human density (secondary axis; for details see Supplementary Materials, Supplementary Table 1). (**b**) Estimates of diet (±95% Bayesian credibility intervals) from mixing models revealed that the contemporary urban interface population had the lowest reliance on native herbivores, while the contemporary wildland population specialized almost entirely upon them. **(c–e)** Isotopic signatures of prey (plotted as corrected standard ellipses) from left to right: native herbivores, large domestic species, synanthropic wildlife, and small domestic species. Cougars (black dots) in the contemporary urban interface possessed the widest niche breadth (standard ellipse; in black). Cougars in the historic urban interface were isotopically distinct from their contemporary counterparts.

Cougars from the three populations (contemporary urban interface, historic urban interface, and contemporary wildlands) differed in isotopic signature (K nearest-neighbour, *p *< 0.001; Fig. 1b–d; Supplementary Table 4). The contemporary urban interface population occupied a broader isotopic niche (SEA_C_ = 1.1; SEA_CB_ = 1.1), compared to both the historic urban interface (SEA_C_ = 0.6; SEA_CB_ = 0.6) and wildland populations (SEA_C_ = 0.7; SEA_CB_ = 0.6), and these differences were significant (contemporary urban vs. wildland: *p* = 0.03; contemporary urban vs. historic urban: *p* = 0.004; Supplementary Fig. 2). We did not detect a difference in isotopic niche size between cougars inhabiting the historic urban interface and wildlands (*p* = 0.44). Bootstrap analysis, as well as analyses of adults only, indicated that the patterns we observed were not driven by outliers, differences in sample sizes, or the demographic composition of samples (Supplementary Materials, Fig. S2, Table S5).

Differences in isotopic niche reflected differences in resource use and dietary diversity between populations, as evidenced by population-wide diet estimates. Contemporary cougars in the urban interface used the highest diversity of prey, with 63–79% of their assimilated biomass from native herbivores (95% Bayesian credibility intervals; Fig. 1e; Supplementary Table S4), and the rest from urban-associated food resources like domestic species (exotics) and synanthropic wildlife (invasives). The wildland population, conversely, relied almost entirely (91–99% of assimilated biomass) on native herbivores (Fig. 1e;), likely large ungulates like elk *Cervus elaphus* (Linnaeus 1758) and mule deer *Odocoileus hemionus* (Rafinesque 1817). We also observed temporal changes in diet; cougars in the urban interface in the 1980s were intermediate in use of native herbivores (Fig. 1e; 73–95% of diet).

## Discussion

Cougars are opportunistic predators, and it appears that this plasticity could be one of the mechanisms by which they successfully exploit novel ecosystems. Indeed, we found that land use changes corresponded with shifts in dietary inputs and overall isotopic niche, and may indicate a changing ecology for cougars in these novel and developing landscapes. Cougars in the wildlands and those in the urban interface 25 years ago relied almost exclusively on native herbivores, principally large bodied ungulates. While still heavily reliant on native ungulates, cougars in the urban interface today interact with a more diverse group of prey species, including both exotic and invasive prey (synanthropic mesocarnivores and domestic species) which are abundant in developed habitats^23^. Though the land use change in the urban interface of Colorado’s Front Range over the past 25 years has primarily consisted of rural-to-exurban transformation (Supplementary Table S2), it appears this intensification of development is associated with large and rapid changes in diet composition for cougar inhabitants.

Shifts in cougar diet over time and space may reflect differences in the availability of prey species, given the higher abundance of exotic and invasive prey in developed habitats^23^. However, there is evidence that cougars may actually select for smaller-bodied prey within developed landscapes to reduce handling time and thus risk^22^. It is highly unlikely, however, that the observed change in diet is simply due to changes in ungulate densities, which have remained relatively constant in the urban interface (Supplementary Materials, Figure S3). Regardless of whether shifts in resource use are due to increased availability of alternative prey or selection for smaller-bodied prey (or both), cougars have demonstrated a shift in resource use in developed areas, across both spatial and temporal scales. Although it is unclear whether this shift is repeated elsewhere, we anticipate this pattern holds true in other developed systems globally given the similarity of these areas regardless of geographic location^1^.

The expansion in diet of cougars has a number of implications for both cougar conservation and the community dynamics of developed systems. For instance, the consumption of domestic species in developed landscapes enhances cougar mortality rates by increasing the risk of conflict with humans^21^, which could represent an ecological trap. Interactions with domestic and synanthropic prey, including closely related species (i.e. wild mesocarnivores and domestic cats and dogs), can also alter disease dynamics due to shared pathogens^24^,^25^. Finally, shifts in resource use by apex carnivores, even if the change is driven by a very few individuals, have the potential to alter the dynamics of prey populations, restructure community assemblages and transform ecosystem functioning^26^,^27^. It remains to be seen how the rapid changes in diet we have observed will affect the relationship between cougar and their ungulate prey, and is an interesting line of future research.

Apex carnivores, which are important members of ecological communities, and are among the most threatened group of species on Earth, are less sensitive to habitat development than previously assumed, and are showing evidence of adaptation to human-dominated landscapes. Our work indicates that development intensification is associated with changes in resource use for one such apex carnivore over the course of only a few decades. Behavioural plasticity is an encouraging sign for carnivore conservation, but could also mean that these species are departing from their historic ecological relationships. Therefore, conserving or reintroducing species to novel urban landscapes will not necessarily resurrect historical ecological interactions, but may create novel ones instead.

## Methods

### Study sites

We evaluated resource use by contemporary (2008–2013) and historic (1983–1990) cougars in an urban interface, as well as contemporary (2008–2013) cougars in a wildland habitat. Within each site, we classified landcover using housing density^28^ and refer to urban, suburban, exurban, and rural lands as “developed” and protected, wildland habitat as “undeveloped”, though developed habitats vary widely in intensity and degree of ecological transformation. The wildland site, on the Uncompahgre Plateau of west-central Colorado, contains little developed habitat (6% of total land-cover), mostly along the perimeter of the study area, all of which constitutes low intensity exurban development (Fig. 1a; Supplementary Materials; Supplementary Table S1; Supplementary Fig. S1). The urban interface site, along the Northern Front Range of Colorado, is one of the major urban-wildland interfaces in the United States^29^. Urban and suburban habitat, which tend to be unsuitable for large carnivores, make up a small fraction (1%) of the study area. A sizeable proportion of the land area is exurban and rural (28% and 14%, respectively); this land use is of particular interest for cougar ecology, as the intermediate intensity of development provides attractive habitat for cougars, yet differs from wildland habitat in community composition and risk factors^18^. Over half (56%) of the urban interface site is undeveloped, and these undeveloped lands are patchy, occurring in close proximity with developed landscapes (Supplementary Fig. S1). Human density is 6× greater than in the wildland site (Fig. 1a). In the 1980s, when historic cougars were sampled, this urban interface had 20% lower human density (Fig. 1a) and was intermediate in habitat development and human density (Supplementary Table S1). Interestingly, between 1980 and 2010, there was almost no conversion of undeveloped lands; rather, development on rural lands intensified, increasing exurban landcover (from 21 to 28%).

### Sampling and isotopic analysis

We collected contemporary samples from cougars during live captures or necropsies; we obtained historic samples from hunter mounts and museum specimens (Supplementary Materials). To estimate cougar diet and niche breadth, we analysed the isotopic signature of cougar and prey tissues in our study areas. Estimates obtained from stable isotopes are not biased towards larger-bodied prey^30^; and therefore can more accurately quantify dietary inputs and niche breadth. We have previously demonstrated^21^ that isotopic signature predicts both where cougars forage and what they forage upon, essential aspects (bionomic and scenopoetic) of a consumer’s occupied ecological niche^31^. Finally, isotopic analysis can be performed on non-invasively collected tissues, making comparisons over broad geographic or temporal scales more feasible. This approach, then, has the power to detect shifts in resource use and realized niche for cryptic, wide-ranging large carnivores at a scale that has previously been impossible.

We captured and sampled hair from 58 adult and sub-adult cougars in the contemporary wildland site and 41 in the contemporary urban interface, from 2008 to 2013 (Supplementary Materials; Supplementary Table S2). All animal handling was in accordance with ACUC 16-2008 and 08-2004 approved by Colorado Parks & Wildlife, Fort Collins, CO. We also collected nine hair samples from the urban interface site between 1983 and 1990, using hunter mounts and museum specimens (Supplementary Materials, Supplementary Table S3). Finally, we collected hair from 17 potential prey species (Supplementary Materials). Hair samples were prepared using standard methods^32^ and analysed for carbon (δ^13^C) and nitrogen (δ^15^N) signature, reported as parts per thousand [‰] ratios relative to standards. We corrected for isotopic discrimination, the enrichment of heavy isotopes at higher trophic levels (Supplementary Materials). We grouped prey into isotopically distinct groups using K nearest-neighbour tests^33^. Prey clustered into four categories: native herbivores, synanthropic mesocarnivores, small domestic animals (pets), and large domestic animals (livestock), representing biologically meaningful classes (Table 1).

To compare the isotopic niche of the three cougar populations, we computed corrected standard ellipse areas (SEA_C_) for each population^34^. Standard ellipses are bivariate estimates of variance in isotopic signature within a population and a useful metric for population-wide niche breadth. To compare ellipse areas between populations, we utilized a bootstrap approach (Supplementary Materials), which also allowed us to test the robustness of our estimates of SEA_C_ to sample size and outliers. We report the median SEA_C_ from bootstrap simulations (SEA_CB_), as well as median p-values from t-tests (Supplementary Fig. S2). To interpret the ecological significance of isotopic niche shifts, we estimated population-wide diet compositions using Bayesian mixing models^35^,^36^. We report 95% credibility intervals of Bayesian posterior probability distributions, which represent the most likely proportion of each diet item for a given population of consumers.

## Additional Information

**How to cite this article**: Moss, W. E. *et al*. Human expansion precipitates niche expansion for an opportunistic apex predator (*Puma concolor*). *Sci. Rep.* **6**, 39639; doi: 10.1038/srep39639 (2016).

**Publisher's note:** Springer Nature remains neutral with regard to jurisdictional claims in published maps and institutional affiliations.

## Supplementary Material

Supplementary Methods, Figures, and Tables

## Figures and Tables

**Table 1:** Isotopic signatures ( 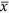 ± SD for potential prey of cougars (*Puma concolor*), collected between 2008 and 2013 in a wildland and an urban interface study area.

Group	*n*	Species	δ^13^C	δ^15^N
Native herbivores (urban interface)	48	Cottontail rabbit (*Sylvilagus nuttallii*)	−21.8 ± 1.0	7.2 ± 2.0
		Mule deer (*Odocoileus hemionus*)		
		Elk (*Cervus elaphus*)		
Native herbivores (wildland)	15	Cottontail rabbit (*Sylvilagus nuttallii*)	−21.5 ± 0.4	8.5 ± 1.1
		Mule deer (*Odocoileus hemionus*)		
		Elk (*Cervus elaphus*)		
Large domestic species	26	Llama (*Lama glama*)	−19.9 ± 1.4	10.3 ± 1.6
		Alpaca (*Vicugna pacos*)		
		Goat (*Capra aegagrus hircus*)		
		Sheep (*Ovis aries*)		
Synanthropic wildlife	38	Striped skunk (*Mephitis mephitis*)	−18.0 ± 1.3	10.8 ± 1.4
		Raccoon (*Procyon lotor*)		
		Fox (*Vulpes vulpes*)		
		Coyote (*Canis latrans*)		
		Squirrel (*Sciurus spp.*)*		
Small domestic species	29	Dog (*Canis familiaris*)	−14.0 ± 2.5	9.6 ± 1.3
		Cat (*Felis catus*)		
		Chicken (*Gallus domesticus*)		

Prey were grouped into isotopically distinct and biologically relevant groups and corrected using isotopic discrimination factors (δ^13^C = +2.6‰; δ^15^N = +3.4‰) so they could be directly compared to cougar signatures. Isotopic signatures for native herbivores differed between study sites.

^*^Not identified to species level.
