# Supplementary material for: Human expansion precipitates niche expansion for an opportunistic apex predator (Puma concolor)
Source: Sci Rep. 2016 Dec 23;6:39639. doi: 10.1038/srep39639 (PMC5180354; doi:10.1038/srep39639)
Supplement: Supplementary Methods, Figures, and Tables [file srep39639-s1.pdf]

Human expansion precipitates niche expansion for an opportunistic apex predator (*Puma concolor*)

Wynne E. Moss[1*], Mathew W. Alldredge[2], Kenneth A. Logan[2], and Jonathan N. Pauli[1]

[1] Department of Forest & Wildlife Ecology, University of Wisconsin, Madison, Wisconsin 53706, USA

[2]Colorado Parks & Wildlife, Fort Collins, Colorado, 80525, USA

*wmoss@wisc.edu

SUPPLEMENTARY INFORMATION

Study areas

We conducted our study in two regions of Colorado, which represent extremes in human influence. The wildland site, located in the Uncompahgre Plateau (Fig. S1), varies in elevation from about 1,700 m to 3,000 m. The most abundant large herbivores in the wildland study area are mule deer (*Odocoileus hemionus*) and elk (*Cervus elaphus*), although sheep and cattle range within the study area during the summer months. Human population density and road density are low, and over half of the land-cover is publicly owned and undeveloped (Table S1). The urban interface site, located in the Northern Front Range (Fig. S1), ranges from 1,600 to 4,300 m in elevation. The south-eastern part of the urban interface site lies within the boundary of the expanding Denver metropolis, while the south-western part of the study area is primarily publicly owned wildland; thus, this study area is a matrix of both developed and undeveloped habitat (Table S1). We sampled cougars within both of these study areas from 2008-2013, as well as cougars living in the urban interface study area in the 1980s, which we refer to as the historic urban interface. In the 1980s, there was 25% less developed habitat and 20% fewer people in the urban interface study area, though the level of habitat development and human density was still higher than observed in the wildland study area. Cougar density in the wildland study site was at least 2.5 independent cougars per 100 km^2;[1], with densities in the contemporary urban interface likely between 2 and 3 (Colorado Parks & Wildlife unpublished data).

We quantified measures of anthropogenic influence within our study areas, as given by housing density, road density, and human population density. We obtained

population counts at the census block group level in the contemporary sites[2] and the historic sites[3]. We selected all the census block groups whose centroid lay within the bounds of the study site and summed their populations. Density was calculated by dividing total population by the area of all selected census block groups. To determine road density, we used TIGER/Line shapefiles[4] to quantify the km of road per km^2 study area.

Housing density and land use categories for historic and contemporary study sites were taken from the SERGoM model of housing density[5]; we used the 1990 historical data layer for the historic urban interface study site and the projected 2010 layers for the contemporary sites. We refer to exurban, suburban, or urban habitat (> 5 units/km^2) as "developed". Undeveloped habitat contained no houses, while rural habitat was between 0.01 and 6 units/km^2.

Finally, to determine if changes in ungulate density were driving changes in diet, we examined mule deer population trends within the urban interface site from 1988 to 2010. Estimates of post-hunt population in the Boulder Creek deer herd (which overlaps with the urban interface study area) were taken from Colorado Parks & Wildlife reports[6]. Mule deer abundance and density did not change in the urban interface since the late 1980s (Fig. S3), therefore, differences in diet over time are unlikely to be the result of changing ungulate availability.

Capture and isotopic sampling

Cougars in the wildland and urban interface site were captured and monitored from 2008-2013 as part of a larger on-going study by Colorado Parks & Wildlife[7,8]. Sub-adult and adult cougars were captured using dogs, cage traps, and snares, and immobilized with

tiletamine hydrochloride (Telazol) or ketamine hydrochloride-medetomidine. Hair samples for isotopic analysis were taken either at captures or necropsies (Table S2). All animal handling was in accordance with ACUC 16-2008 and 08-2004 approved by Colorado Parks & Wildlife, Fort Collins, CO. We searched state records for cougars harvested within our study area between 1970 and 1990 and requested samples from hunters. We also queried museum databases for samples within 50 km of our study area prior to 1990 (Table S3).

We collected over 140 hair samples from over 15 prey species, which we identified as being potentially important due to their prevalence at kill sites within our study area or from previous studies of cougar diet[9,10]. Hair samples from wild prey species were collected at cougar kill sites or road kills in both study areas. We sampled domestic species in the wildland-urban study site using shed hairs from farms or veterinary clinics, and assumed that domestic species would not vary geographically due to a high reliance on commercial feed rather than wild plants. We did not sample prey from the 1980s in the urban interface site, but assumed that prey isotopic signature did not change over time.

Isotopic analysis

Hair samples from cougar and prey were rinsed in a 2:1 mixture of chloroform: methanol, dried for 72 hours, and homogenized, following standard methods[11]. Samples were analysed using a Carlo Erba 1100 Elemental Analyzer coupled to a Thermo Delta Plus XP IRMS. Results are reported as parts per thousand [‰] ratios relative to international standards of Peedee Belemnite (PDB; $\delta^{13}C$) and atmospheric nitrogen (AIR; $\delta^{15}N$). We

adjusted prey isotopic signatures using isotopic correction factors for carnivores ($\delta^{13}C$ = +2.6 ‰; $\delta^{15}N$ = +3.4; [12]). After correction, we grouped prey into biologically relevant and isotopically distinct source groups using a K nearest-neighbour randomization test[13]. Synanthropic wildlife from the wildland and urban interface sites did not differ (K nearest-neighbour; $p > 0.05$); however, the native herbivore group differed in $\delta^{15}N$ among study areas ($p < 0.01$). Therefore, with the exception of the native herbivore group, we used identical prey isotopic signatures in mixing models for all three populations. The native herbivore group contains rabbits, elk, and deer, but, because ungulates are an order of magnitude larger, use of this prey group reflects mostly consumption of ungulates, rather than small mammals.

We corrected the $\delta^{13}C$ signatures of historic cougar samples to account for the Suess effect, or the decrease in atmospheric ^{13}C from fossil fuel burning[14]. We applied a -0.022 ‰ per year correction[15]. To compare differences in raw isotopic signature between cougar populations, we used K nearest-neighbour tests.

We computed a corrected standard ellipse (SE_C) for each population in the program SIAR[16], with the area of each ellipse (SEA_C) representing amount of isotopic variation within a population. Compared with convex hull or standard deviation methods, estimates of SEA_C are more robust to differences in sample size and are, therefore, a useful measure of niche breadth when sample sizes differ among groups, as in our study[17].

However, because standard ellipses are, themselves, estimates of uncertainty[18], the area of an ellipse does not have an associated variance estimate. In order to derive an estimate of variance and test the robustness of SEA_C estimates to sample sizes and

outliers, we utilized a bootstrapping approach. First, given the small sample size of the historic urban interface population (*n* = 9), we tested the sensitivity of SEA_C estimates to outliers. We calculated SEA_C after excluding one sample, and repeated this nine times, dropping a different sample each time. SEA_C varied little, from 0.4 – 0.7, suggesting no individual sample was overly influential (Fig. S2). Next, we used a similar process for the contemporary urban and contemporary wildland datasets. Nine individuals were randomly drawn from each sample set, and SEA_C was estimated for those nine individuals. We repeated this sampling nine times, to generate a distribution similar to the one generated for the historic urban interface (Fig. S2). We compared each simulated distribution with the historic urban interface distribution using a Welch's t-test. We also compared the contemporary wildland and contemporary urban distributions to one another. We repeated this process 1000×, and calculated an average p-value and median SEA_C (Fig. S2).

Though SEA_C provides a useful estimate of niche *breadth*, we could not directly compare niche *overlap* (as measured by SE_C overlap in bi-plots) between cougar populations because the position of the SE_C can be influenced by variation in prey signature among groups, and may not necessarily reflect differences in foraging strategy [18]. Thus, we estimated diet compositions for populations in SIAR, which allowed us to compare niche overlap independent of differences in the isotopic signature of prey, as in Flaherty and Ben-David[19]. To estimate diet, SIAR uses Markov Chain Monte Carlo simulations to generate a distribution of possible diets that are consistent with consumer and prey isotopic signatures[15]. The output is given as Bayesian posterior distributions of possible solutions[20]. We report diet as 95% Bayesian credibility intervals of these

distributions, which represents the most likely diet for an entire population. We grouped small and large domestic species *a posteriori* because, though they differed isotopically, the model could not accurately distinguish between these two sources.

Finally, to test whether differences between study areas were influenced by the demographic composition of samples, we ran analyses using only adults from the two contemporary populations. Because adults are more likely than subadults to consume large-bodied native herbivores[21], it is possible that differences in the demographic structure of the three sample sets (Table S2) could influence estimates of native herbivore use. Restricting the analysis to adults only in the contemporary population is the most conservative approach, given that adults have the most ungulate specialized diets. We retained subadults and unknown age individuals (n = 3) in the historic urban interface population, because if anything, this would increase our estimate of alternative prey and thus dietary breadth. With this new analysis, adults in the contemporary urban interface still had broader isotopic niches and relied less on native herbivores than the historic population (Table S5), and estimates of diet and niche breadth varied only slightly. Similarly, restricting analyses to adults only in the wildland population also did not change results significantly (Table S5). Thus, the patterns still held true, with isotopic niche the largest and reliance on native herbivores the lowest in contemporary urban interface cougars. Therefore, to maximize sample sizes and avoid confusion, we report full results from analyses of the entire sample sets.

146

Tables

Table S1. Measures of anthropogenic influence in Colorado study areas where we sampled cougars (*Puma concolor*). The wildland study site shows the lowest amount of anthropogenic influence and the contemporary urban interface study site the highest. In the 1980s, the urban interface site was intermediate for all measures of anthropogenic influence. Land use estimates and classifications were derived from the SERGoM model of housing density[5] and are given as % area.

	Wildland (contemporary)	Urban interface (historic)	Urban interface (contemporary)
Human density (persons/km^{2})	7	33	41
Road density (km roads/km^{2})	1.1	*	2.3
**Land use (% total area)**			
Undeveloped	72	58	56
Rural	22	19	14
Exurban	6	21	28
Suburban	< 1	1	1
Urban	< 1	< 1	< 1
Total area (km^{2})	2898	2869	2869

*Digitized maps of roads during the 1980s were not available for our study area.

Table S2. Age-sex classes of cougars sampled in contemporary wildland and urban interface study sites. Ages were determined using Logan's[21] criteria. Adults were individuals > 24 months who had established a home range and sub-adults were independent individuals < 24 months old.

Study site	Age-sex class	*N*	% of total sample
Wildland	Adult female	24	41
	Adult male	11	19
	Sub-adult female	9	16
	Sub-adult male	14	24
Contemporary urban interface	Adult female	21	51
	Adult male	7	17
	Sub-adult female	7	17
	Sub-adult male	6	15
Historic urban interface	Adult female	2	22
	Adult male	4	44
	Sub-adult (unknown sex)	1	11
	Unknown	2	22

Table S3. Historic samples collected from the urban interface study area in Colorado. Samples were obtained from museums or from mounts. We report accession numbers for museum samples.

Source	Mortality	Age-sex class	Year	Accession number
Mount	Harvested	Adult male	1983	
Mount	Harvested	Adult male	1983	
Mount	Harvested	Adult male	1986	
Mount	Roadkill	Subadult female	1989	
Mount	Harvested	Adult female	1986	
Mount	Harvested	Adult male	1983	
Denver Museum of Nature & Science	Unknown	Unknown female	1988	DMN ZM.7699
Museum of Southwestern Biology	Agency	Adult female	1988	MSB:111939
Museum of Southwestern Biology	Unknown	Unknown female	1990	MSB: 115606

Table S4. Isotopic signatures and diet estimates for three cougar (*Puma concolor*) populations in Colorado. Dietary estimates are derived from Bayesian mixing models; we give the mean and 95% Bayesian credibility interval (CI) from simulations to estimate diet. The wildland population shows the highest reliance on native herbivores, followed by the historic urban interface population. The contemporary urban interface population relies most heavily upon alternative prey species.

			Isotopic signature: $\bar{x} \pm$ SD		Dietary contribution: $\bar{x}$ (95% CI)		
Study Area	Time	*n*	δ^{13}C (‰)	δ^{13}N (‰)	Native herbivores	Domestic species	Synanthropic wildlife
Wildland	Contemporary (2008-2013)	58	-21.6 ± 0.5	8.5 ± 0.5	96% (91-99%)	4% (1-8%)	1% (0-3%)
Urban interface	Historic (1983-1990)	7	-21.6 ± 0.5	6.9 ± 0.6	85% (73-95%)	11% (1-21%)	5% (0-12%)
Urban interface	Contemporary (2008-2013)	41	-21.3 ± 0.65	8.1 ± 0.8	71% (63-79%)	23% (13-33%)	5% (0-12%)

Table S5. Results of analysis to test the impacts of demographic (i.e. subadult vs. adult) representation in samples. We restricted contemporary samples to adults only to enable direct comparisons of diet and niche breadth, and to reduce possible effects of differences in demographic structure of samples. We compared contemporary adult cougars to the full sample of historic cougars, which included subadult individuals. Our analysis demonstrated that patterns of niche expansion and dietary shifts were robust to the demographic classes sampled.

	Full population analysis		Adults-only analysis	
Population	Dietary contribution from native herbivores*	Isotopic niche (SEA_C)	Dietary contribution from native herbivores*	Isotopic niche (SEA_C)
Contemporary urban interface	63–79%	1.1	65–84%	1.0
Contemporary wildland	84–98%	0.7	91–99%	0.6
Historic urban interface	73–95%	0.6	N/A	N/A

*95% Bayesian credibility interval

Figures

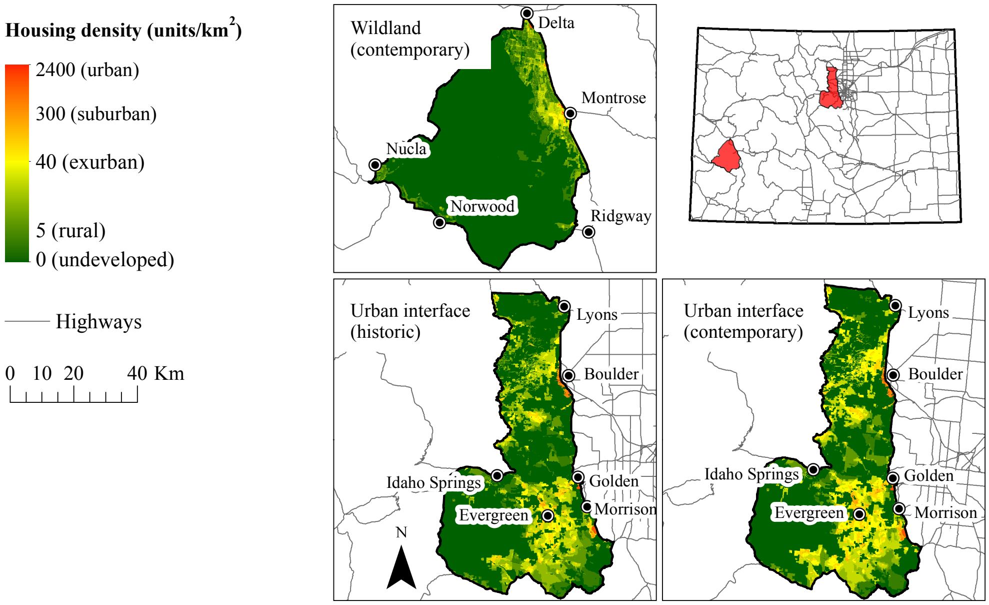

Figure S1. Study areas in Colorado, where we sampled cougars (*Puma concolor*) for isotopic analysis. The wildland study site, which was sampled from 2008 – 2013, is located on the Uncompahgre Plateau of west-central Colorado, which has little anthropogenic influence. The Northern Front Range of Colorado is an expanding urban-wildland interface; we sampled this site in the 1980s (urban interface historic) and from 2008-2013 (urban interface contemporary). Housing density is classified with SERGoM housing density raster layers[5] using 2010 data for contemporary land use and 1990 data for historic land use. Maps were created using ArcGIS software and base maps (Version 10.2, Esri, www.esri.com).

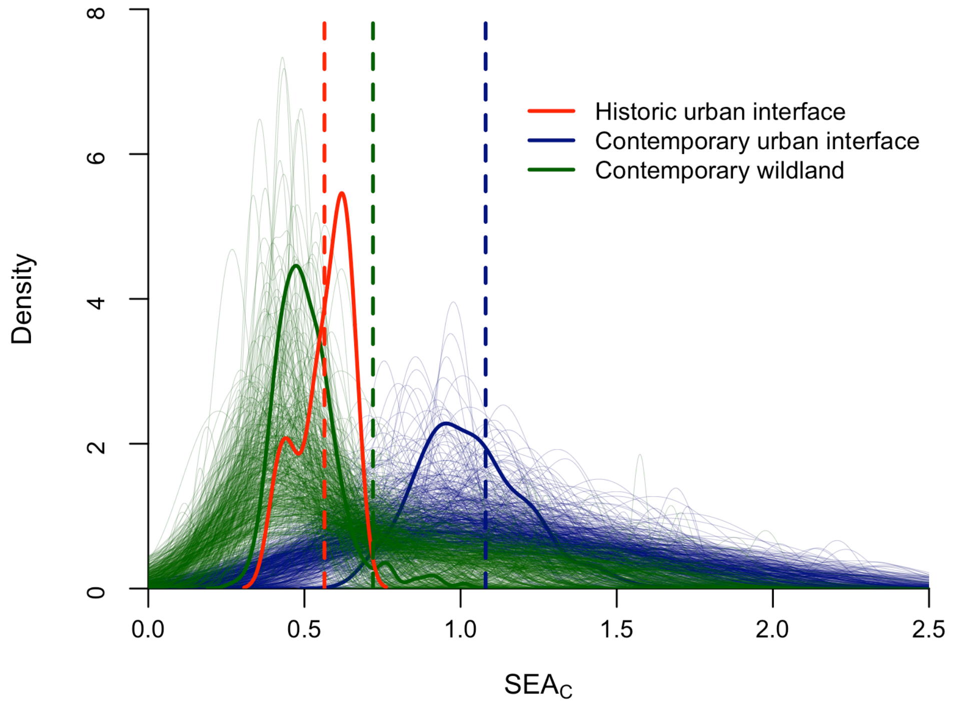

Figure S2. Bootstrapped comparison of SEA_C (corrected standard ellipse areas) between cougar (*Puma concolor*) populations. For the contemporary urban interface and wildland populations, we randomly drew nine individuals from the larger sample set and calculated SEA_C; this was repeated nine times to give a distribution of SEA_C estimates (a single thin line). We repeated this 1000 times and plotted the median distribution (heavy line). For the historic urban interface population (*n* =9), we randomly dropped one sample and calculated SEA_C for the remaining samples, and repeated this for all possible combinations, giving one distribution (in red). Non-bootstrapped estimates of SEA_C (dashed lines) are also shown.

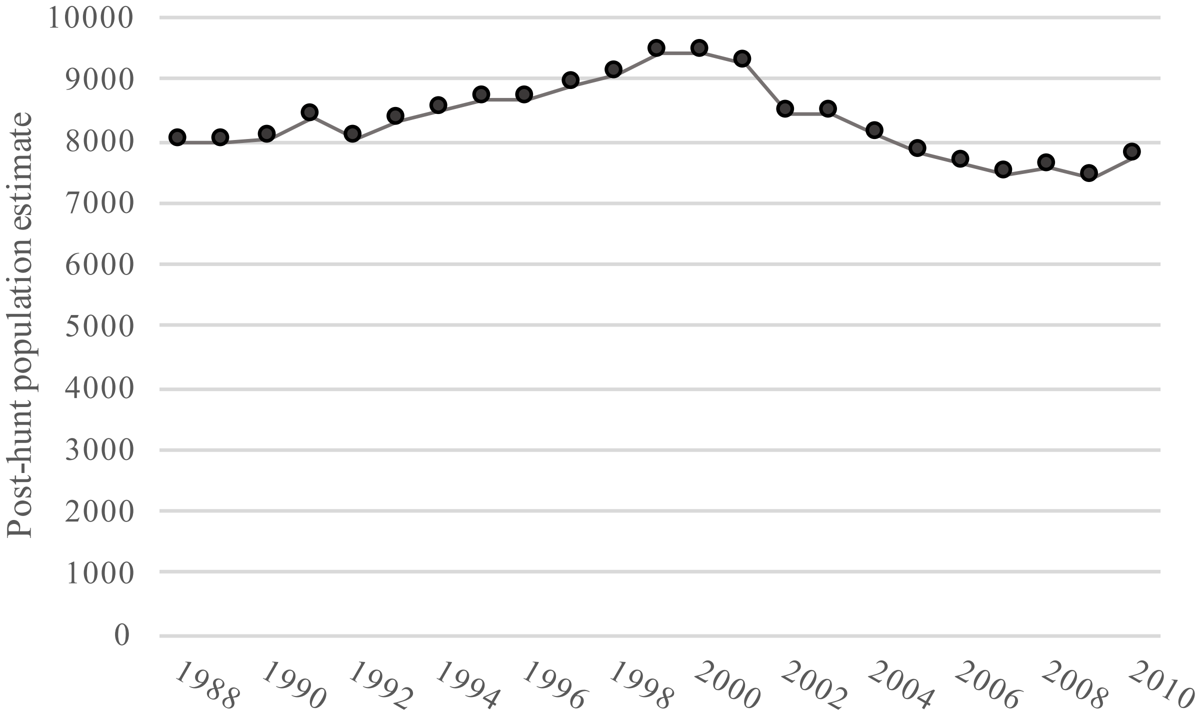

Figure S3. Estimates of population size for the deer herd in the Boulder Creek Deer Herd management unit . The urban interface study area is located within this management unit. Estiamtes are modeled post-hunt populations, gathered by Colorado Parks & Wildlife[6]. Deer abundance was near 8000 for both historic (1980s) and contemporary (2007-2013) sampling periods, although pre-1988 estimates were not available.

References

1. Logan, K. A. Puma population structure and vital rates on the Uncompahgre Plateau. *Annual Report, Mammals Program, Colorado Division of Parks and Wildlife* (2013).

2. United States Census Bureau. Census of population and housing: summary tape file 1 on CD ROM (Colorado). (2010). https://www.nhgis.org.

3. United States Census Bureau. Census of population and housing: summary tape file 1 on CD ROM (Colorado). (1990). https://www.nhgis.org.

4. United States Census Bureau. TIGER/Line Mapping Service: Colorado state. (2010). www.census.gov/geo/maps-data/data/tiger-line.html.

5. Theobald, D. M. Landscape patterns of exurban growth in the USA from 1980 to 2020. *Ecol. Soc.* **10,** 32 (2005).

6. Huwer, S. & Kraft, B. D-27 Boulder Creek deer herd management plan. *Colorado Parks and Wildlife* (2012).

7. Alldredge, M. W. Cougar demographics and human interactions along the urban-exurban Front-Range of Colorado. *Annual Report, Mammals Research, Colorado Parks and Wildlife* (2011).

8. Logan, K. A. Puma population structure and vital rates on the Uncompahgre Plateau. *Annual Report, Mammals Research, Colorado Parks and Wildlife* (2009).

9. Kertson, B. N., Spencer, R. D. & Grue, C. E. Cougar prey use in a wildland–urban environment in western Washington. *Northwest. Nat.* **92,** 175–185 (2011).

10. Knopff, K. H., Knopff, A. A., Kortello, A. & Boyce, M. S. Cougar kill rate and prey composition in a multiprey system. *J. Wildl. Manage.* **74,** 1435–1447 (2010).

11. Pauli, J. N., Ben-David, M., Buskirk, S. W., DePue, J. E. & Smith, W. P. An isotopic technique to mark mid-sized vertebrates non-invasively. *J. Zool.* **278,** 141–148 (2009).

12. Roth, J. D. & Hobson, K. A. Stable carbon and nitrogen isotopic fractionation between diet and tissue of captive red fox: implications for dietary reconstruction. *Can. J. Zool.* **78,** 848–852 (2000).

13. Rosing, M. N., Ben-David, M. & Barry, R. P. Analysis of stable isotope data: a K nearest-neighbors randomization test. *J. Wildl. Manage.* **62,** 380–388 (1998).

14. Sonnerup, R. E. *et al*. Reconstructing the oceanic 13C Suess Effect. *Global Biogeochem. Cycles* **13,** 857–872 (1999).

15. Hopkins, J. B. & Ferguson, J. M. Estimating the diets of animals using stable isotopes and a comprehensive Bayesian mixing model. *PLoS One* **7,** e28478 (2012).

16. Parnell, A., Inger, R., Bearhop, S. & Jackson, A. L. SIAR: stable isotope analysis in R. (2008).

17. Jackson, A. L., Inger, R., Parnell, A. C. & Bearhop, S. Comparing isotopic niche widths among and within communities: SIBER - Stable Isotope Bayesian Ellipses in R. *J. Anim. Ecol.* **80,** 595–602 (2011).

18. Newsome, S. D., del Rio, C. M., Bearhop, S. & Phillips, D. L. A niche for isotopic ecology. *Front. Ecol. Environ.* **5,** 429–436 (2007).

19. Flaherty, E. A. & Ben-David, M. Overlap and partitioning of the ecological and isotopic niches. *Oikos* **119,** 1409–1416 (2010).

20. Parnell, A. C., Inger, R., Bearhop, S. & Jackson, A. L. Source Partitioning Using Stable Isotopes: Coping with Too Much Variation. *PLoS One* **5,** (2010).

20. Moss, W. E., Alldredge, M. W. & Pauli, J. N. Quantifying risk and resource use for a large carnivore in an expanding urban–wildland interface. *J Appl Ecol* **53**, 371–378 (2016).

21. Logan, K. A. & Sweanor, L. L. *Desert puma: evolutionary ecology and conservation of an enduring carnivore*. (Island Press, 2001).
